# Superficial peroneal nerve entrapment in ankle sprain in childhood and adolescence

**DOI:** 10.1038/s41598-021-94647-x

**Published:** 2021-07-23

**Authors:** Francesco Falciglia, Luca Basiglini, Angelo G. Aulisa, Renato M. Toniolo

**Affiliations:** grid.414125.7 0000 0001 0727 6809 U.O.C. of Orthopedics and Traumatology, Bambino Gesù Children’s Hospital, Institute of Scientific Research, P.zza S. Onofrio 4, 00165 Rome, Italy

**Keywords:** Musculoskeletal system, Nervous system, Physical examination, Chronic pain, Paediatric research, Trauma

## Abstract

Traumatic injuries of the ankle are the most common injuries in sports. Up to 40% of patients who have undergone inversion ankle sprain report residual symptoms. The primary purpose of the study is to evaluate the incidence of SPN entrapment as consequence of acute severe inversion ankle sprain in children and adolescents; the secondary is to report the diagnostic pathway and the results after surgical treatment. From 2000 to 2015 were reviewed to summarize patients under the age of 15 years treated for a first episode of severe inversion ankle sprain. Cases with persistent symptoms (more than 3 months) indicative for SPN neuropathy were then identified. Instrumental investigations were recovered and a pre-operative assessment of pain (VAS) was recorded. Patients were evaluated at minimum of 1-year post-operative follow-up. 981 acute ankle sprains have been evaluated. 122 were considered severe according to van Dijk criteria. 5 patients were considered affected by neuropathy of the SPN. All patients underwent surgery consisting in neurolysis and capsular retention and ligament reconstruction. At 25 months of follow-up AOFAS moved from 57.6 to 98.6. The study highlights a previously unreported condition of perineural fibrosis of the superficial peroneal nerve at the level of the ankle following first acute severe inversion ankle sprain in children.

## Introduction

**Figure 1:**
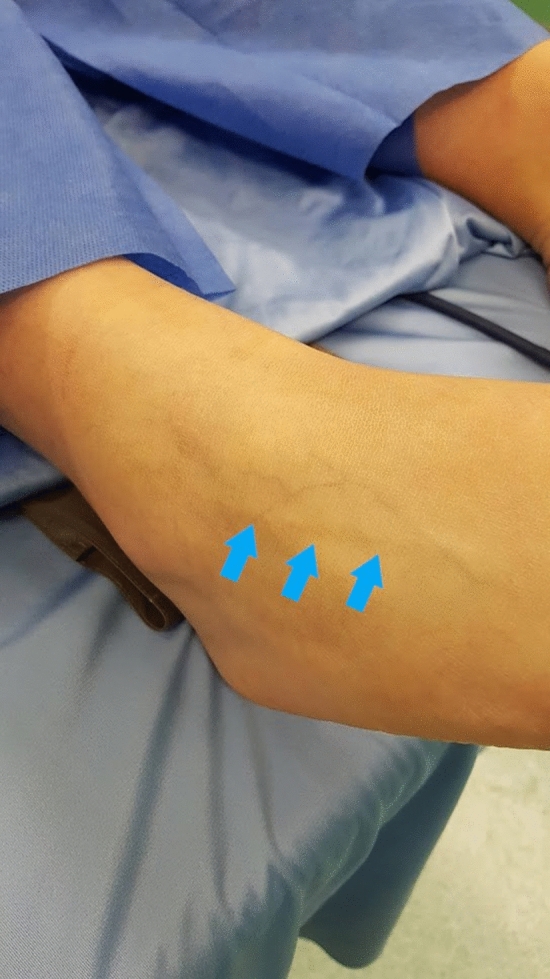
Clinical picture of the course of the superficial peroneal nerve at ankle joint level in preoperative assessment.

Traumatic injuries of the ankle are the most common injuries in sports^1^ and the most frequently encountered in the treatment of traumatic injuries of young athletes. Up to 40% of patients who have undergone inversion ankle sprain report residual symptoms^2^. In childhood this could be related to several factors such as the severity of the sprain, an occult osteochondral avulsion, residual mechanical instability or nerve injuries. The superficial peroneal nerve (SPN) is a lateral branch of the common peroneal nerve; it begins at the neck of the fibula and runs through the peroneal muscles. It courses through the intermuscular septum between the anterior and lateral leg compartments and through the deep fascia that is situated in the subcutaneous tissue of the lower third of the leg. SPN supplies sensation to the foot’s dorsal skin; it is divided in two branches, the intermediate medial cutaneous nerve and the intermediate dorsal cutaneous nerve which may be involved in ankle sprain (Fig. 1)^3^. Peroneal nerve palsy is a well-known complication following varus knee injuries or fibula head fractures^4^. On the other hand, less well known is the lesion of the branches of the common peroneal nerve following an inversion ankle sprain. Formerly, these distraction lesions have been described only at the popliteal fossa and fibular neck levels. Although Kenzora et al.^5^ described surgical injuries to the intermediate dorsal cutaneous nerve, traction injuries to this branch of the superficial peroneal nerve have not been widely identified. In literature we have not found papers with evaluations of cases with chronic neuropathy due to involvement of the superficial peroneal nerve (SPN) in the growing age. The primary aim of the study is to evaluate the incidence of SPN entrapment as consequence of acute severe inversion ankle sprain in children and adolescents; the secondary aim is to report the diagnostic pathway and the results after surgical treatment.

## Materials and methods

Office records from 2000 to 2015 were reviewed to summarize patients under the age of 15 years treated for a first episode of severe inversion ankle sprain. Clinical examination demonstrated a history of acute pain with sensation of “crack”, marked limited function, remarkable swelling and pain evoked by palpation in the lateral compartment. Severity of the injury was clinically evaluated according to Van Dijk^6^. Radiographs showed no evidence of fractures.

Cases with persistent symptoms (more than 3 months) indicative for SPN neuropathy were then identified. Clinical signs included: pain, tingling, numbness and paresthesia at the dorsum of the foot; a positive Tinel-like sign was in all cases were just anterior to the lateral malleolus. Moreover, instrumental investigations (X-ray and MRI) were recovered and a pre-operative assessment of pain (VAS) was recorded. Operative reports were reviewed to evaluate surgical findings. Patients were evaluated at 7 days, 1 months and at minimum of 1 year post-operative follow-up using AOFAS (American Orthopedic Foot and Ankle Society) score.

### Inclusion criteria

Patients with complete clinical data and affected by first acute severe ankle sprain with negative X-ray conservatively treated.

### Exclusion criteria

Incomplete clinical records, previous ankle surgery or other surgical treatment in the involved leg. Congenital malformations and connective tissue disorders.

### Ethical approval

Ethical approval was waived by the local Ethics Committee of Bambino Gesù Children’s Hospital in view of the retrospective nature of the study and all the procedures being performed were part of the routine care. All methods were carried out in accordance with relevant guidelines and regulations.

### Consent to participate

Written informed consent was obtained from the parents.

### Consent to publish

Written informed consent for publication of their clinical details and clinical images was obtained from the parents. A copy of the consent form is available for review by the Editor of this journal.

## Results

In our emergency department 981 acute ankle sprain has been accepted in 15 years. Among them 122 were considered severe according to van Dijk criteria with negative X-ray for fractures or osteochondral avulsion. 5 patients were considered affected by neuropathy of the SPN: the patients were two boys and three girls ranging in age from 8 to 13 years (average 10.6 years). All patients experienced a first episode inversion ankle sprain resulting from sport trauma in three cases (2 volleyball and 1 basketball); one trauma was consequent from a fall from the stairs and one patient hurt while running. They were all treated with RICE: rest, ice, compression and elevation therapy to reduce swelling of the ankle and foot for 5 days and non-weight-bearing with crutches for 14 days. Physical therapy concentrating on proprioception and peroneal strengthening was performed after the period of non-weight bearing.

Although severe swelling and acute pain resolved at a short-term follow-up (1 month), they continued to have a neuritic type pain at the dorsolateral aspect of the foot. They all had a positive percussion sign over the intermediate dorsal cutaneous branch of the superficial peroneal nerve (Fig. 1) just anterior to the lateral malleolus (Tinel-like sign). Drawer test and varus stress were positive in all the patients compared with the controlateral ankle. Given the persistence of the symptomatology, the patients underwent II level instrumental examinations (MRI) which showed no lesions evident with the method except outcomes of distracting alterations of the anterior talo-fibular ligament (ATFL). In the first three patients we approached a conservative treatment with local anesthetic infiltration on the Tinel-like sign area. After the infiltration we prescribed a cast and light-bearing for 15 days. Although all patients had a transient resolution of the symptoms, the neuritic symptomatology recurred in one month time. On these bases, patients were taken to surgery. In the other two patients, 3 months after the trauma, was directly proposed a surgical treatment decompression due to the failure of conservative treatment. All patients underwent surgery performed by the same senior surgeon (FF).

### Surgical findings

**Figure 2:**
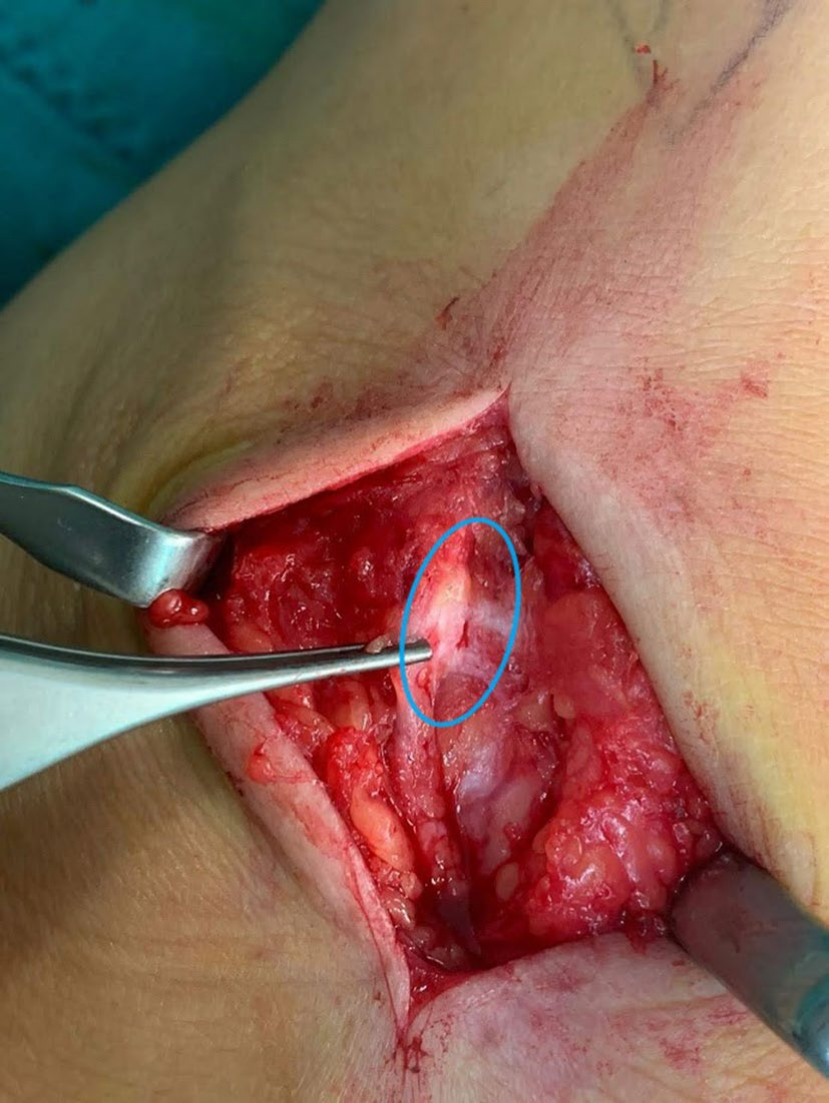
Surgical exposure of superficial peroneal nerve with region of perineural fibrosis.

**Figure 3:**
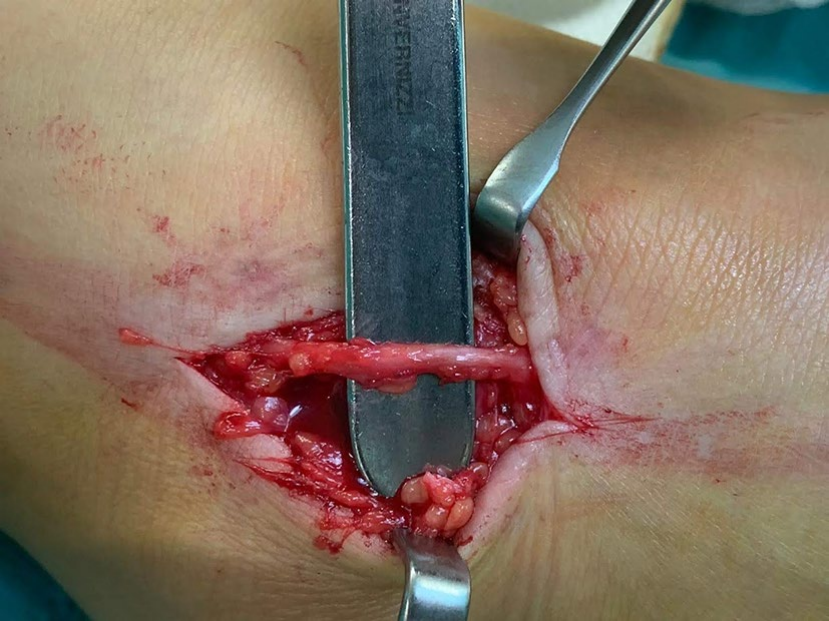
Surgical exposure of superficial peroneal nerve after neurolysis and medial transposition into subcutaneous fat. All the photos in Figs. 1, 2 and 3 were taken by the same senior surgeon (FF).

All patients were operated on under general anesthesia. With the patient in supine position, surgeon performed a 5–8 cm skin incision in line with the anterior fibula, just anterior to the preoperative Tinel-like sign (marked in preoperative area). After fascia incision, the intermediate dorsal cutaneous branch of the superficial peroneal nerve was exposed at the level of the lateral malleolus and traced proximally to the anterior border of the fibula in the interval between the peroneal muscles and the extensor digitorum longus. An abnormal nerve anatomy was noted in each patient: the nerve was incorporated into the reparative scar of the previous sprain (Fig. 2). Dissecting the nerve and releasing it from the scar and from any tethering soft tissue allowed to translate the nerve anteromedially into subcutaneous fat, until full ankle motion was achieved without increased nerve tension (Fig. 3). In order to definitely stabilize the joint, capsular retention and ligament reconstruction was then performed with a portion of extensor retinaculum (Broström modified surgical technique). Closure of the wound did not include the approximation of the crural fascia, performing only a single-layer closure in order to minimize the risk of recurrence.

No perioperative complication was highlighted. All patients reported disappearance of the Tinel-like sign. All patients were evaluated at an average follow-up of 25 months. All of them reported a marked improvement in symptoms, moving from an AOFAS preoperative score of 57.6 to a postoperative of 98.6. They all returned to the sporting and / or competitive level prior to the trauma (Table 1).

**Table 1:** Summary table of patients surgically treated for superficial peroneal nerve entrapment.

Name	Age	Gender	Side	Trauma	Operative findings	AOFAS pre	AOFAS post	F.U. (months)
LC	11	F	L	Running	Infiltration + neurolysis + capsuloplasty	82	100	24
AM	8	M	L	Stairs	Infiltration + neurolysis + capsuloplasty	62	100	49
SM	13	F	R	Volley	Infiltration + neurolysis + capsuloplasty	71	100	20
ML	11	F	R	Volley	Neurolysis + capsuloplasty	43	97	15
GA	10	M	L	Basket	Neurolysis + capsuloplasty	30	96	17

## Discussion

Superficial peroneal nerve entrapment is relatively rare. Identification and treatment of entrapment of the SPN are important topics of discussion for orthopedic surgeons because overlooking the diagnosis can lead to permanent nerve damage. Donovan and colleagues^7^ stated that if the symptoms of nerve entrapment persist for 2 to 3 months, then surgical decompression is usually required to prevent permanent nerve damage. To avoid this, early diagnosis and early intervention are very important^8^. As a matter of fact, this is not always easy given the devious clinical manifestation of the neuritic pain. Several studies in the literature have related the entrapment of the SPN with different causes such as ankle sprain, ganglion cysts, direct trauma, muscle herniation or mass effect from swelling of tumors (lipomas, schwannomas)^4,9^.

Although many authors, in the literature, recommend early surgical treatment to avoid nerve injury, our results show that full recovery can be achieved even after 3 months. Therefore, before making surgical indication, a conservative treatment can be tried.

We focused on this type of pathology as the result of severe ankle trauma. O’Neill and colleague in their biomechanical study on cadaver specimens highlighted that SPN is particularly vulnerable to stretch from an inversion mechanism because of its anteromedial position when the foot is inverted and plantar flexed^1^ (Fig. 3); they compared the SPN strain of the specimens group with intact ATFL and with dissected ligament, showing that in the sectioned-ligament group there was a significantly higher nerve strain compared with the intact-ligament group. This suggest that the SPN is at risk with ankle sprain and is at additional risk with more severe sprain or with an insufficient ATFL^1^.

In literature the incidence of SPN entrapment in ankle sprain (with negative X-ray) in children is not reported. We defined the incidence of 4% in the first acute severe ankle sprain in children. In case of severe sprain the complete tear of ATFL, capsule and soft tissue cause a reparative scar around the intermediate dorsal cutaneous nerve branch of the SPN which passes just near the ATFL above the fibular malleolus. Scar hypertrophy can cause the entrapment of the above nerve. In literature we found that SPN can be easily subjected to entrapment as it penetrates deep fascia of leg^10^. Moreover, older patients may be predisposed to stretch-type nerve injuries; Nobel found that young cadavers had 10 to 25 mm of stretch in the SPN, whereas old subject tore the nerve at its exit from the fascia near the ankle^11^. Our data show that, although there is greater tolerability to the nerve elongation mechanisms in younger patients, the SPN can be damaged by a reparative scar.

Most patients report pain during activity in the lateral region of the leg and dysesthesias in the dorsolateral region of the foot. We can evoked pain by inverting and plantarflexing the ankle and by tapping the nerve as it emerges from the deep fascia^10^. The results of the history and the physical examination help to understand the problem. NCV studies and EMG are unreliable. Instead radiographs may assist in differential diagnosing of malalignment or instability. The only procedure that can confirm the diagnosis is the relief of pain after a localized injection of anesthetic at the site of Tinel-like^12^.

The symptoms present in our cases were paresthesia, hyposensitivity and pain during plantar flexion and varo-supination. Positive Tinel’s test just pushing on ATFL above the peroneal malleolus. Moreover, drawer and varus tests were positive.

Dissecting the nerve from the scar and releasing it where exit the deep fascia allowed an anteromedially translation of the SPN, allowing ankle motion without putting tension on the nerve. Furthermore, all the patient underwent Broström modified technique to obtain joint stability^13,14^.

Debridement and neurolysis at about 3 months after trauma associated to Broström modified technique lead to good long term results although symptoms improved just after surgery^15^.

This report illustrates a previously unreported condition of perineural fibrosis of the superficial peroneal nerve at the level of the ankle following first acute severe inversion ankle sprain in children. Perineural fibrosis should be considered in the differential diagnosis of patients with persistent pain after ankle sprain and neurolysis should be considered associated to Broström modified technique to obtain stability and release of pain.

## Data Availability

All authors declare that all data and materials support their published claims and comply with field standards.

## Abbreviations

SPN: Superficial peroneal nerve
MRI: Magnetic resonance imaging
VAS: Visual analogue scale
AOFAS: American Orthopedic Foot and Ankle Society
ATFL: Anterior talo-fibular ligament
NCV: Nerve conduction velocity
EMG: Electromyography
